# Supplementary material for: A Systematic Review and Meta-Analysis of Mean Platelet Volume and Platelet Distribution Width in Patients with Obstructive Sleep Apnoea Syndrome
Source: Biomedicines. 2024 Jan 24;12(2):270. doi: 10.3390/biomedicines12020270 (PMC10887137; doi:10.3390/biomedicines12020270)
Supplement: Supplementary file 1 [file biomedicines-12-00270-s001.zip › biomedicines-2789501-supplementary.pdf]

**Full search strategy**

**PubMed**

("OSAS"[All Fields] OR "OSA"[All Fields] OR "obstructive sleep apnea syndrome"[All Fields]) AND ("complete blood count"[All Fields] OR "CBC"[All Fields] OR "full blood count"[All Fields] OR "FBC"[All Fields] OR "mean platelet volume"[All Fields] OR "MPV"[All Fields] OR "platelet distribution width"[All Fields] OR "PDW"[All Fields])

**Web of Science**

("OSAS" OR "OSA" OR "obstructive sleep apnea syndrome") AND ("complete blood count" OR "CBC" OR "full blood count" OR "FBC" OR "mean platelet volume" OR "MPV" OR "platelet distribution width" OR "PDW") (All Fields)

**Scopus**

TITLE-ABS-KEY (( "OSAS" OR "OSA" OR "obstructive sleep apnea syndrome" ) AND ( "complete blood count" OR "CBC" OR "full blood count" OR "FBC" OR "mean platelet volume" OR "MPV" OR "platelet distribution width" OR "PDW" ))

**Suppl. Table S1.** The Joanna Briggs Institute critical appraisal checklist for the included studies.

| Study | Were the criteria for inclusion clearly defined? | Were the subjects and the setting described in detail? | Was the exposure measured in a valid and reliable way? | Were objective, standard criteria used for measurement of the condition? | Were confounding factors identified? | Were strategies to deal with confounding factors stated? | Were the outcomes measured in a valid and reliable way? | Was appropriate statistical analysis used? | Risk of bias |
|---|---|---|---|---|---|---|---|---|---|
| 1. Varol E | yes | yes | yes | yes | yes | yes | yes | yes | low |
| 2. Varol E | yes | yes | yes | yes | no | no | yes | yes | low |
| 3. Nena E | yes | yes | yes | yes | yes | yes | yes | yes | low |
| 4. Karakaş MS | yes | yes | yes | yes | no | no | yes | yes | low |
| 5. Kanbay A | yes | yes | yes | yes | yes | yes | yes | yes | low |
| 6. Kurt OK | yes | yes | yes | yes | no | no | yes | yes | low |
| 7. Sökücü SN | yes | yes | yes | yes | no | no | yes | yes | low |
| 8. Akyüz A | yes | yes | yes | yes | yes | yes | yes | yes | low |
| 9. Gunbatar H | yes | yes | yes | yes | no | no | yes | yes | low |
| 10. Sökücü SN | yes | yes | yes | yes | no | no | yes | yes | low |
| 11. Koseoglu HI | yes | yes | yes | yes | yes | yes | yes | yes | low |
| 12. Yousef AM | yes | yes | yes | yes | yes | yes | yes | yes | low |
| 13. Bülbül Y | yes | yes | yes | yes | no | no | yes | yes | low |
| 14. Song YJ | yes | yes | yes | yes | yes | yes | yes | yes | low |
| 15. Uygur F | yes | yes | yes | yes | no | no | yes | yes | low |
| 16. Hong SN | yes | yes | yes | yes | no | no | yes | yes | low |
| 17. Sarioglu N | yes | yes | yes | yes | yes | yes | yes | yes | low |
| 18. Yilmaz Avci A | yes | yes | yes | yes | yes | yes | yes | yes | low |
| 19. Kıvanc T | yes | yes | yes | yes | no | no | yes | yes | low |
| 20. Archontogeorgis K | yes | yes | yes | yes | no | no | yes | yes | low |
| 21. Fan Z | yes | yes | yes | yes | yes | yes | yes | yes | low |
| 22. Azab NY | yes | yes | yes | yes | yes | yes | yes | yes | low |
| 23. Lakadamyali H | yes | yes | yes | yes | yes | yes | yes | yes | low |
| 24. Ulusoy B | yes | yes | yes | yes | no | no | yes | yes | low |
| 25. Geçkil AA | yes | yes | yes | yes | no | no | yes | yes | low |
| 26. Zorlu D | yes | yes | yes | yes | yes | yes | yes | yes | low |
| 27. Şeyhanlı ES | yes | yes | yes | yes | no | no | yes | yes | low |
| 28. Zota IM | yes | yes | yes | yes | yes | yes | yes | yes | low |
| 29. Geçkil AA | yes | yes | yes | yes | no | no | yes | yes | low |
| 30. Duksal F | yes | yes | yes | yes | no | no | yes | yes | low |
| 31. Yang J | yes | yes | yes | yes | yes | yes | yes | yes | low |

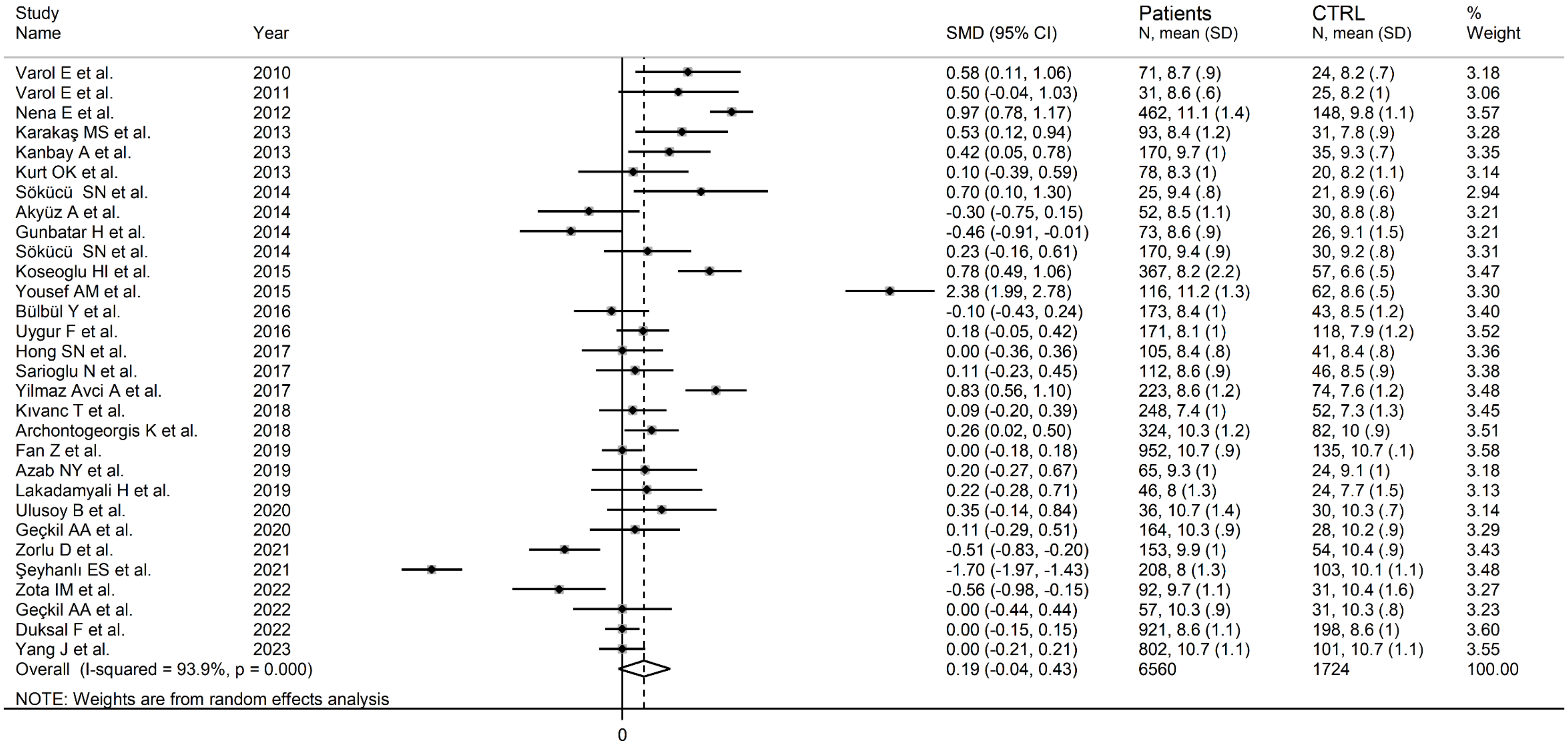


**Suppl. Figure S1**. Forest plot of studies examining MPV values of OSAS patients and controls.

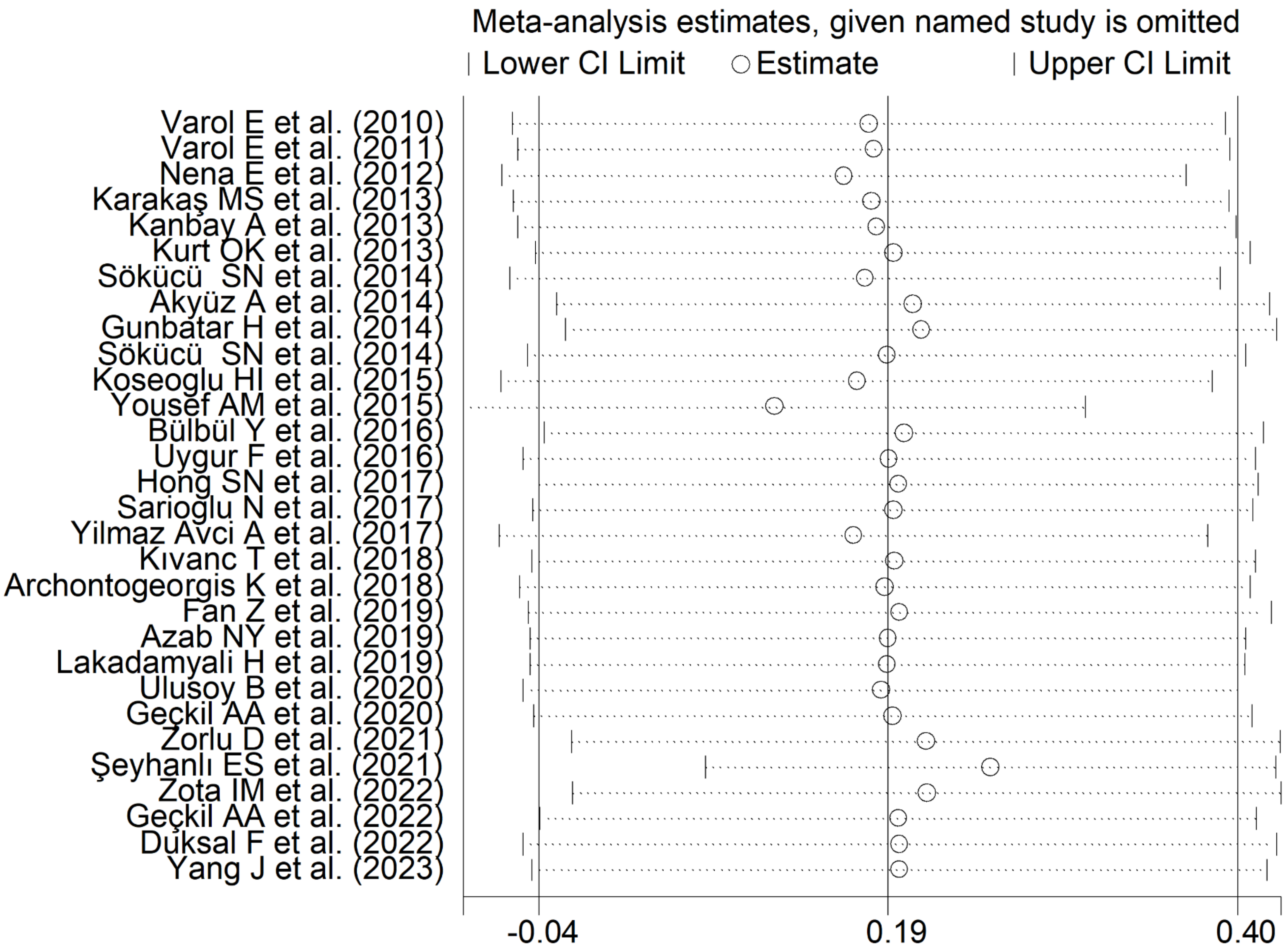


**Suppl. Figure S2.** Sensitivity analysis of the association between MPV values and OSAS. For each study, the displayed effect size (hollow circles) corresponds to an overall effect size computed from a meta-analysis excluding that study.

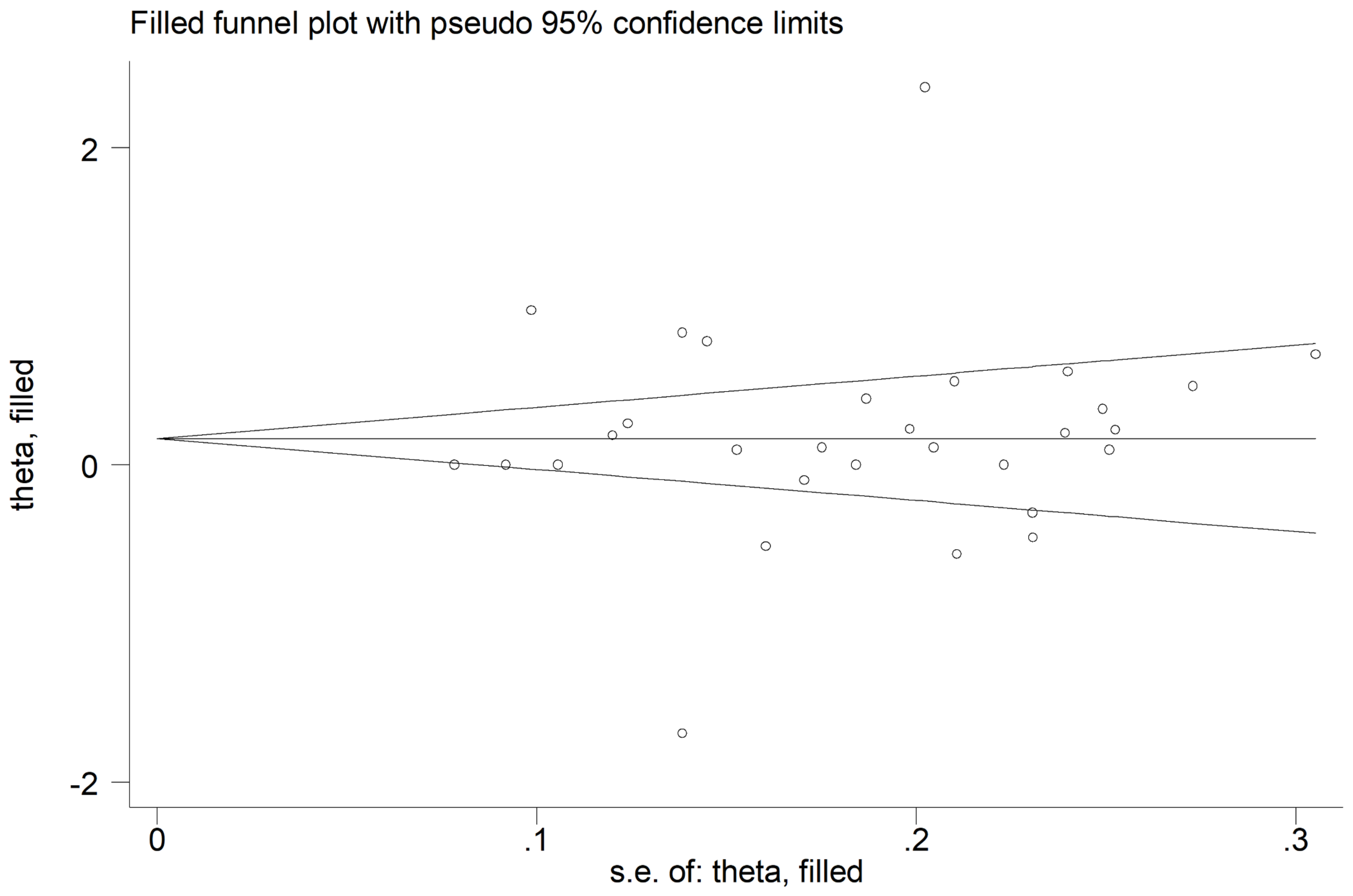


**Suppl. Figure S3.** Funnel plot of studies investigating association between MPV values and OSAS after trimming and filling. Dummy studies and genuine studies are represented by enclosed circles and free circles, respectively.

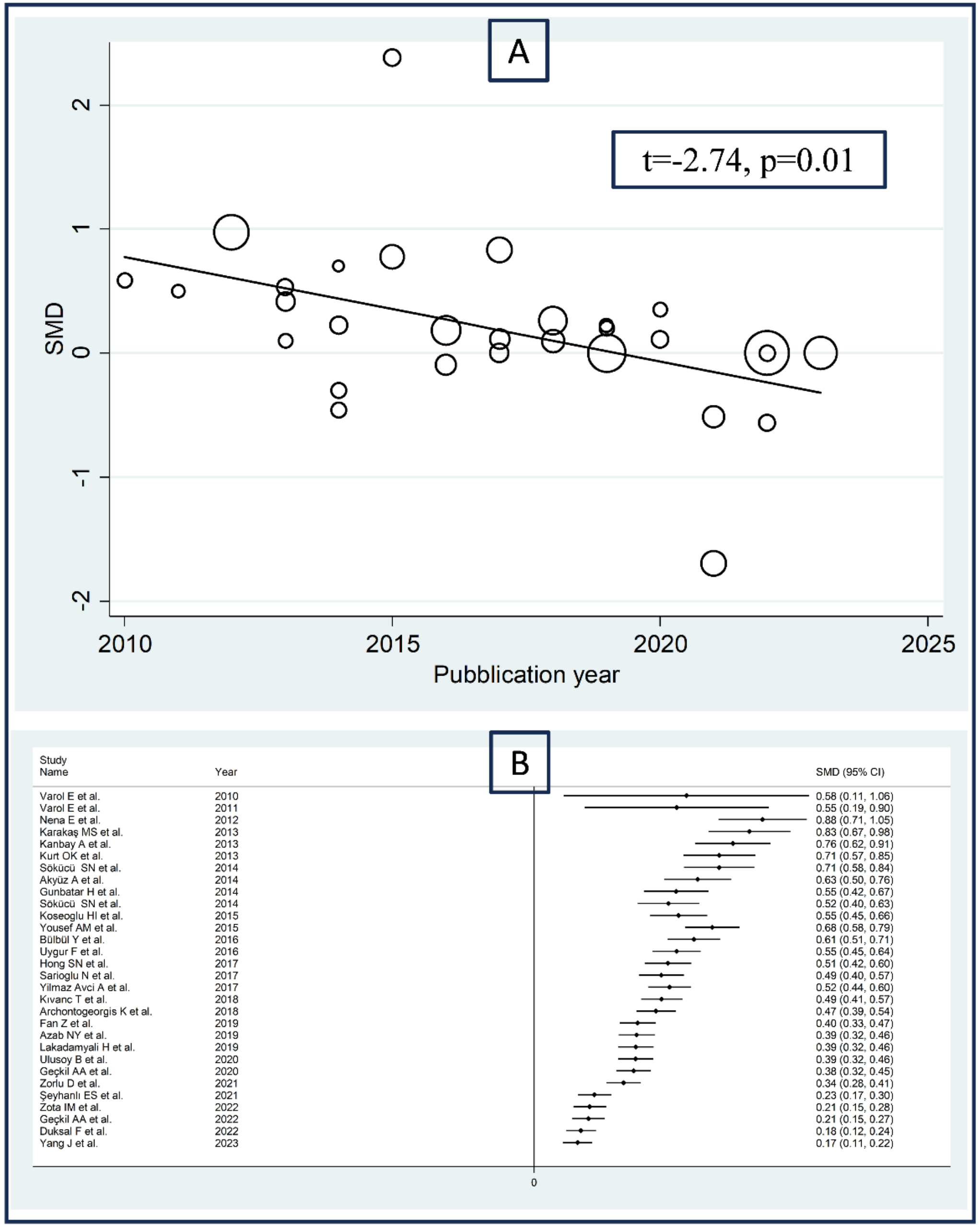


**Suppl. Figure S4.** Bubble plot reporting univariate meta-regression analysis between effect size and publication year (A) and cumulative meta-analysis of MPV values based on publication year (B).

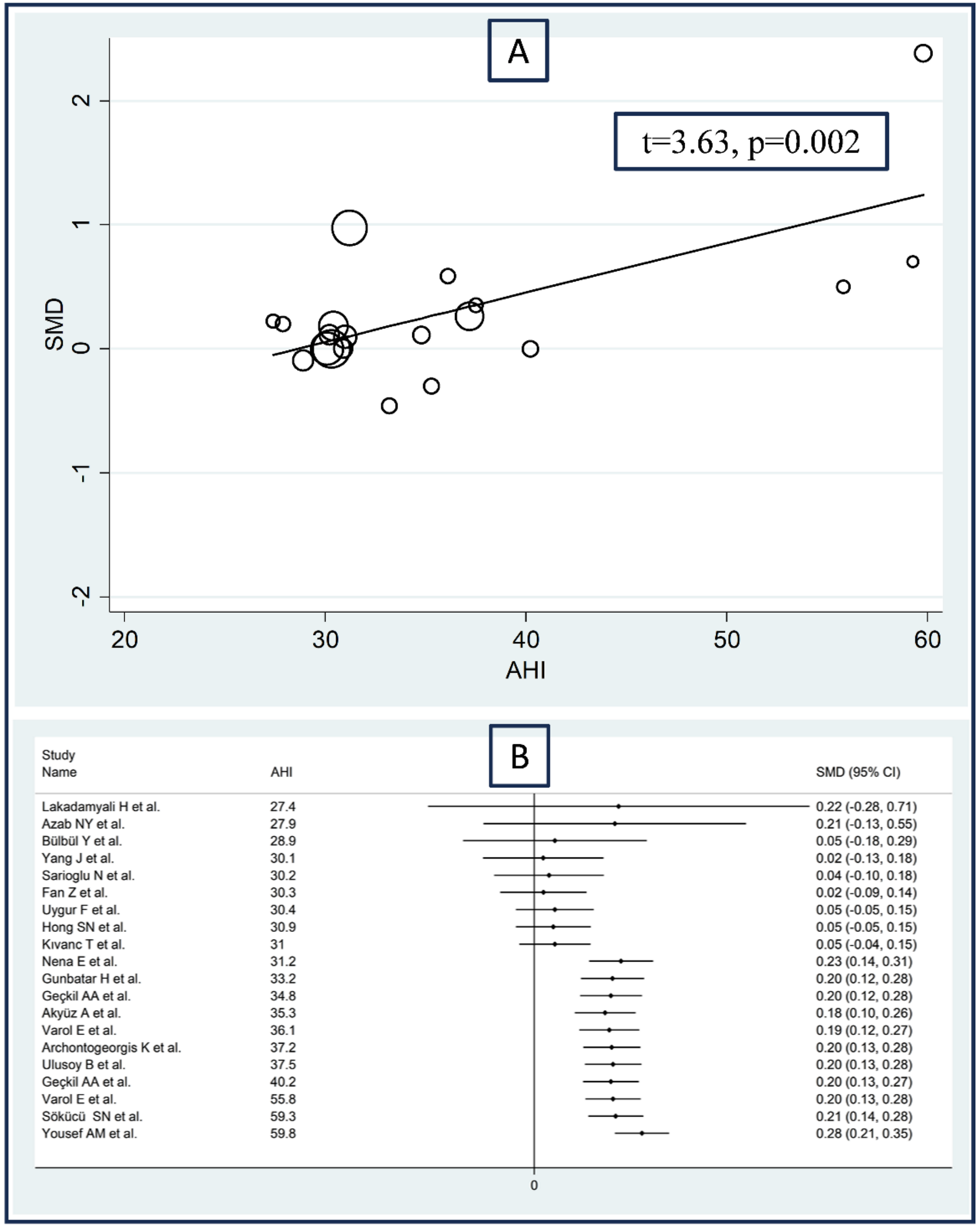


**Suppl. Figure S5.** Bubble plot reporting univariate meta-regression analysis between effect size and AHI (A) and cumulative meta-analysis of MPV values based on AHI (B).

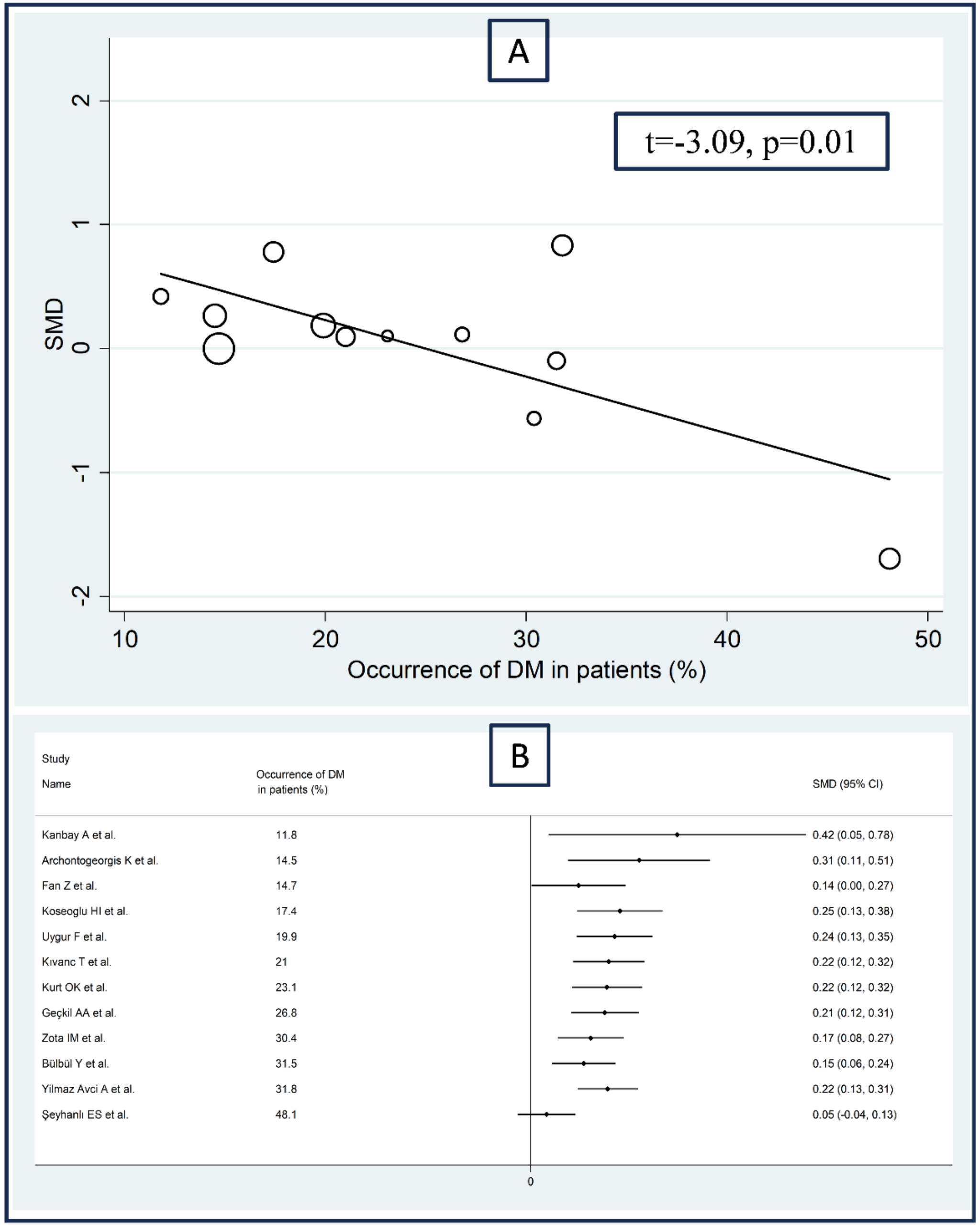


**Suppl. Figure S6.** Bubble plot reporting univariate meta-regression analysis between effect size and occurrence of DM in patients (A) and cumulative meta-analysis of MPV values based on occurrence of DM in patients (B).

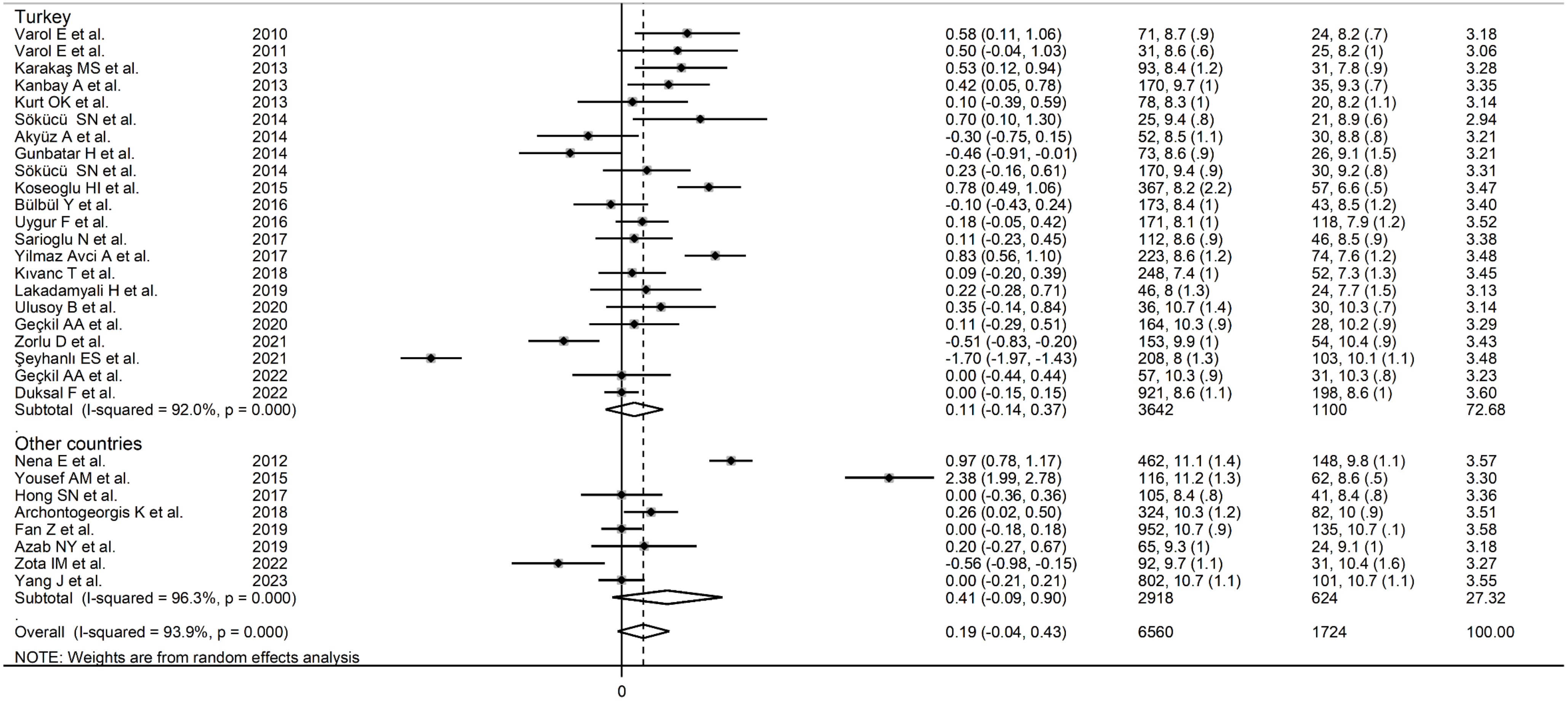

| Study Name | Year | SMD (95% CI) | Patients N, mean (SD) | CTRL N, mean (SD) | % Weight |
|---|---|---|---|---|---|
| **Turkey** | | | | | |
| Varol E et al. | 2010 | 0.58 (0.11, 1.06) | 71, 8.7 (.9) | 24, 8.2 (.7) | 3.18 |
| Varol E et al. | 2011 | 0.50 (-0.04, 1.03) | 31, 8.6 (.6) | 25, 8.2 (1) | 3.06 |
| Karakaş MS et al. | 2013 | 0.53 (0.12, 0.94) | 93, 8.4 (1.2) | 31, 7.8 (.9) | 3.28 |
| Kanbay A et al. | 2013 | 0.42 (0.05, 0.78) | 170, 9.7 (1) | 35, 9.3 (.7) | 3.35 |
| Kurt OK et al. | 2013 | 0.10 (-0.39, 0.59) | 78, 8.3 (1) | 20, 8.2 (1.1) | 3.14 |
| Sökücü SN et al. | 2014 | 0.70 (0.10, 1.30) | 25, 9.4 (.8) | 21, 8.9 (.6) | 2.94 |
| Akyüz A et al. | 2014 | -0.30 (-0.75, 0.15) | 52, 8.5 (1.1) | 30, 8.8 (.8) | 3.21 |
| Gunbatar H et al. | 2014 | -0.46 (-0.91, -0.01) | 73, 8.6 (.9) | 26, 9.1 (1.5) | 3.21 |
| Sökücü SN et al. | 2014 | 0.23 (-0.16, 0.61) | 170, 9.4 (.9) | 30, 9.2 (.8) | 3.31 |
| Koseoglu HI et al. | 2015 | 0.78 (0.49, 1.06) | 367, 8.2 (2.2) | 57, 6.6 (.5) | 3.47 |
| Bülbül Y et al. | 2016 | -0.10 (-0.43, 0.24) | 173, 8.4 (1) | 43, 8.5 (1.2) | 3.40 |
| Uygur F et al. | 2016 | 0.18 (-0.05, 0.42) | 171, 8.1 (1) | 118, 7.9 (1.2) | 3.52 |
| Sarioglu N et al. | 2017 | 0.11 (-0.23, 0.45) | 112, 8.6 (.9) | 46, 8.5 (.9) | 3.38 |
| Yilmaz Avci A et al. | 2017 | 0.83 (0.56, 1.10) | 223, 8.6 (1.2) | 74, 7.6 (1.2) | 3.48 |
| Kıvanc T et al. | 2018 | 0.09 (-0.20, 0.39) | 248, 7.4 (1) | 52, 7.3 (1.3) | 3.45 |
| Lakadamyali H et al. | 2019 | 0.22 (-0.28, 0.71) | 46, 8 (1.3) | 24, 7.7 (1.5) | 3.13 |
| Ulusoy B et al. | 2020 | 0.35 (-0.14, 0.84) | 36, 10.7 (1.4) | 30, 10.3 (.7) | 3.14 |
| Geçkil AA et al. | 2020 | 0.11 (-0.29, 0.51) | 164, 10.3 (.9) | 28, 10.2 (.9) | 3.29 |
| Zorlu D et al. | 2021 | -0.51 (-0.83, -0.20) | 153, 9.9 (1) | 54, 10.4 (.9) | 3.43 |
| Şeyhanlı ES et al. | 2021 | -1.70 (-1.97, -1.43) | 208, 8 (1.3) | 103, 10.1 (1.1) | 3.48 |
| Geçkil AA et al. | 2022 | 0.00 (-0.44, 0.44) | 57, 10.3 (.9) | 31, 10.3 (.8) | 3.23 |
| Duksal F et al. | 2022 | 0.00 (-0.15, 0.15) | 921, 8.6 (1.1) | 198, 8.6 (1) | 3.60 |
| Subtotal (I-squared = 92.0%, p = 0.000) | | 0.11 (-0.14, 0.37) | 3642 | 1100 | 72.68 |
| . | | | | | |
| **Other countries** | | | | | |
| Nena E et al. | 2012 | 0.97 (0.78, 1.17) | 462, 11.1 (1.4) | 148, 9.8 (1.1) | 3.57 |
| Yousef AM et al. | 2015 | 2.38 (1.99, 2.78) | 116, 11.2 (1.3) | 62, 8.6 (.5) | 3.30 |
| Hong SN et al. | 2017 | 0.00 (-0.36, 0.36) | 105, 8.4 (.8) | 41, 8.4 (.8) | 3.36 |
| Archontogeorgis K et al. | 2018 | 0.26 (0.02, 0.50) | 324, 10.3 (1.2) | 82, 10 (.9) | 3.51 |
| Fan Z et al. | 2019 | 0.00 (-0.18, 0.18) | 952, 10.7 (.9) | 135, 10.7 (.1) | 3.58 |
| Azab NY et al. | 2019 | 0.20 (-0.27, 0.67) | 65, 9.3 (1) | 24, 9.1 (1) | 3.18 |
| Zota IM et al. | 2022 | -0.56 (-0.98, -0.15) | 92, 9.7 (1.1) | 31, 10.4 (1.6) | 3.27 |
| Yang J et al. | 2023 | 0.00 (-0.21, 0.21) | 802, 10.7 (1.1) | 101, 10.7 (1.1) | 3.55 |
| Subtotal (I-squared = 96.3%, p = 0.000) | | 0.41 (-0.09, 0.90) | 2918 | 624 | 27.32 |
| . | | | | | |
| Overall (I-squared = 93.9%, p = 0.000) | | 0.19 (-0.04, 0.43) | 6560 | 1724 | 100.00 |

NOTE: Weights are from random effects analysis

**Suppl. Figure S7.** Forest plot of studies examining MPV values of OSAS patients and controls according to country.

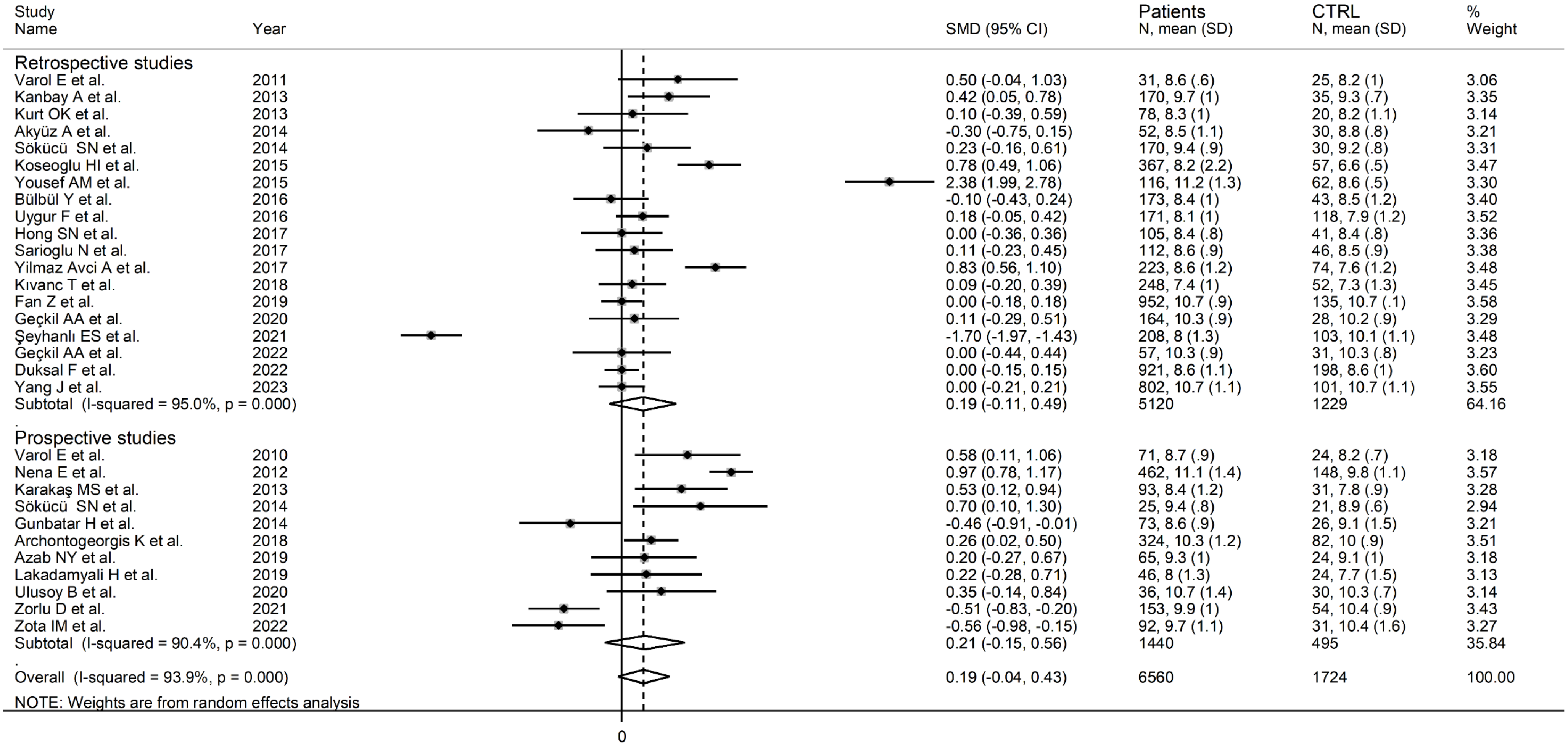


**Suppl. Figure S8.** Forest plot of studies examining MPV values of OSAS patients and controls according to study design.

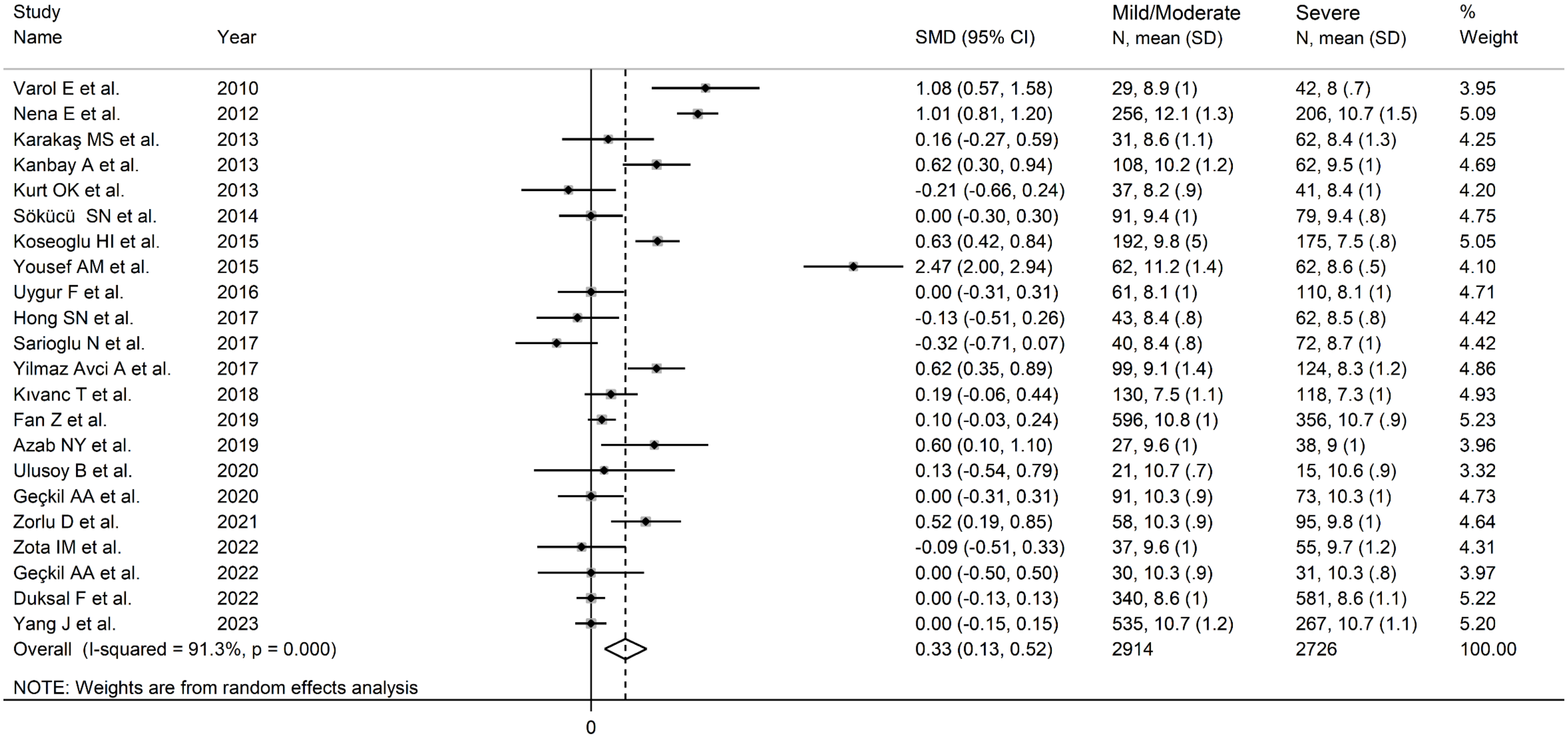


**Suppl. Figure S9.** Forest plot of studies examining MPV values of mild/moderate vs. severe OSAS patients.

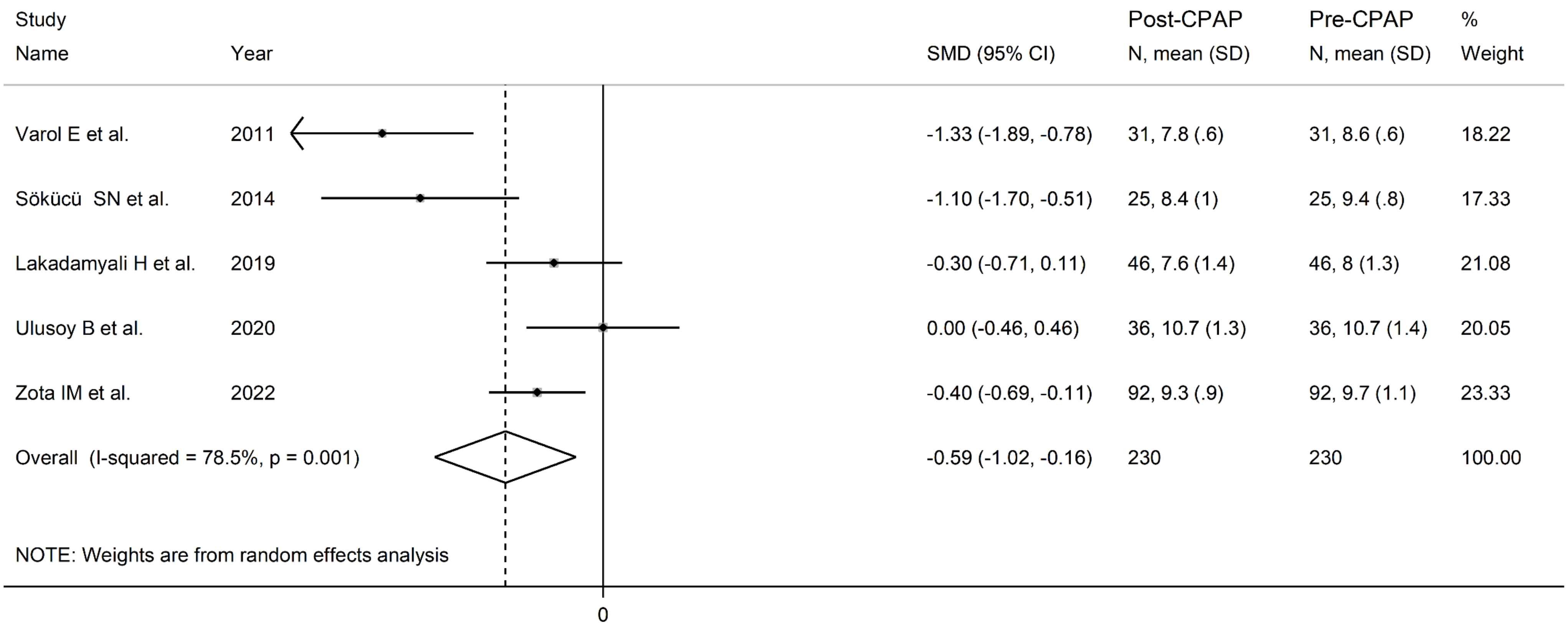


**Suppl. Figure S10.** Forest plot of studies examining MPV values of pre- and post-CPAP treatment patients.

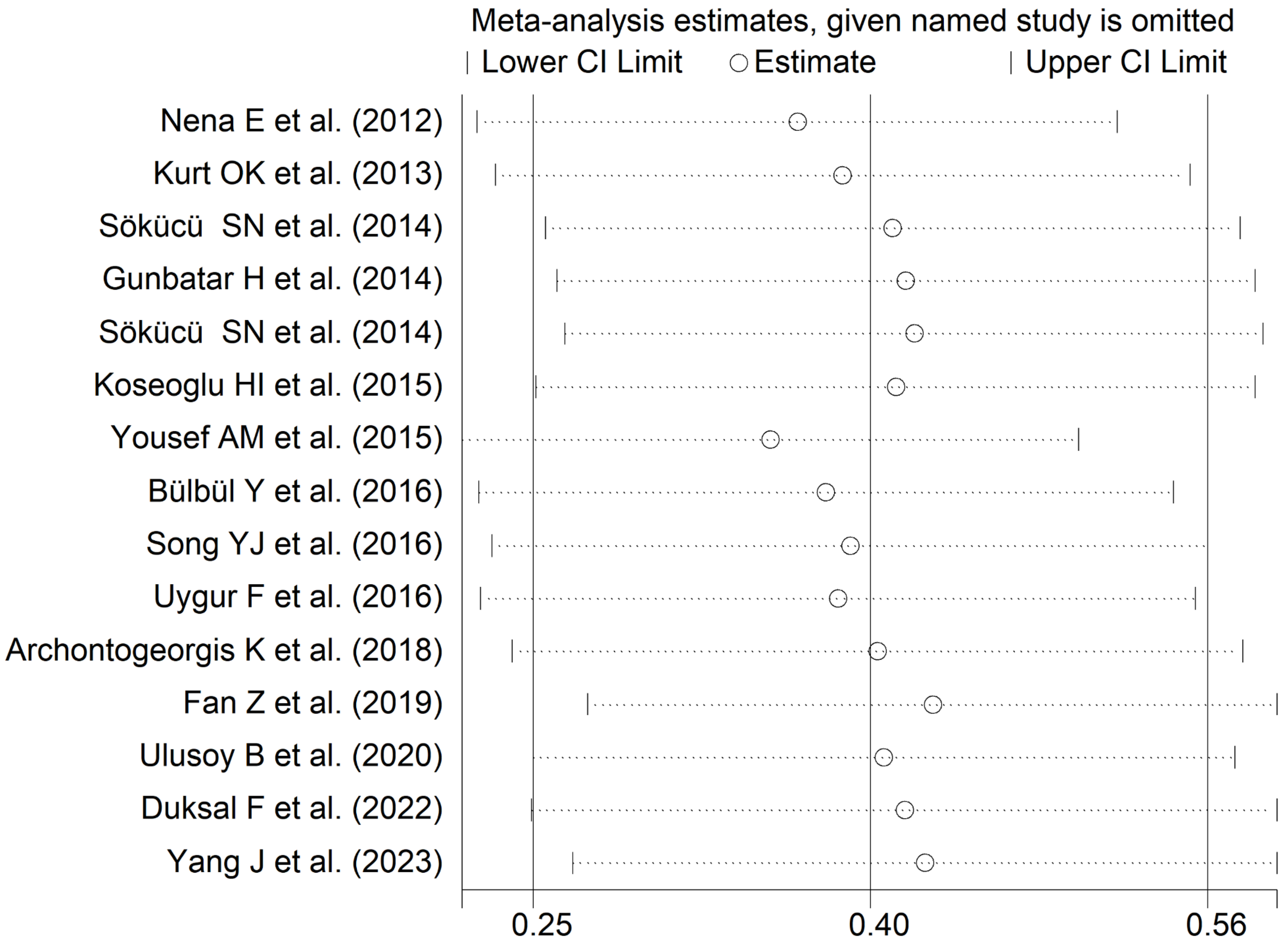


**Suppl. Figure S11**. Sensitivity analysis of the association between PDW values and OSAS. For each study, the displayed effect size (hollow circles) corresponds to an overall effect size computed from a meta-analysis excluding that study.

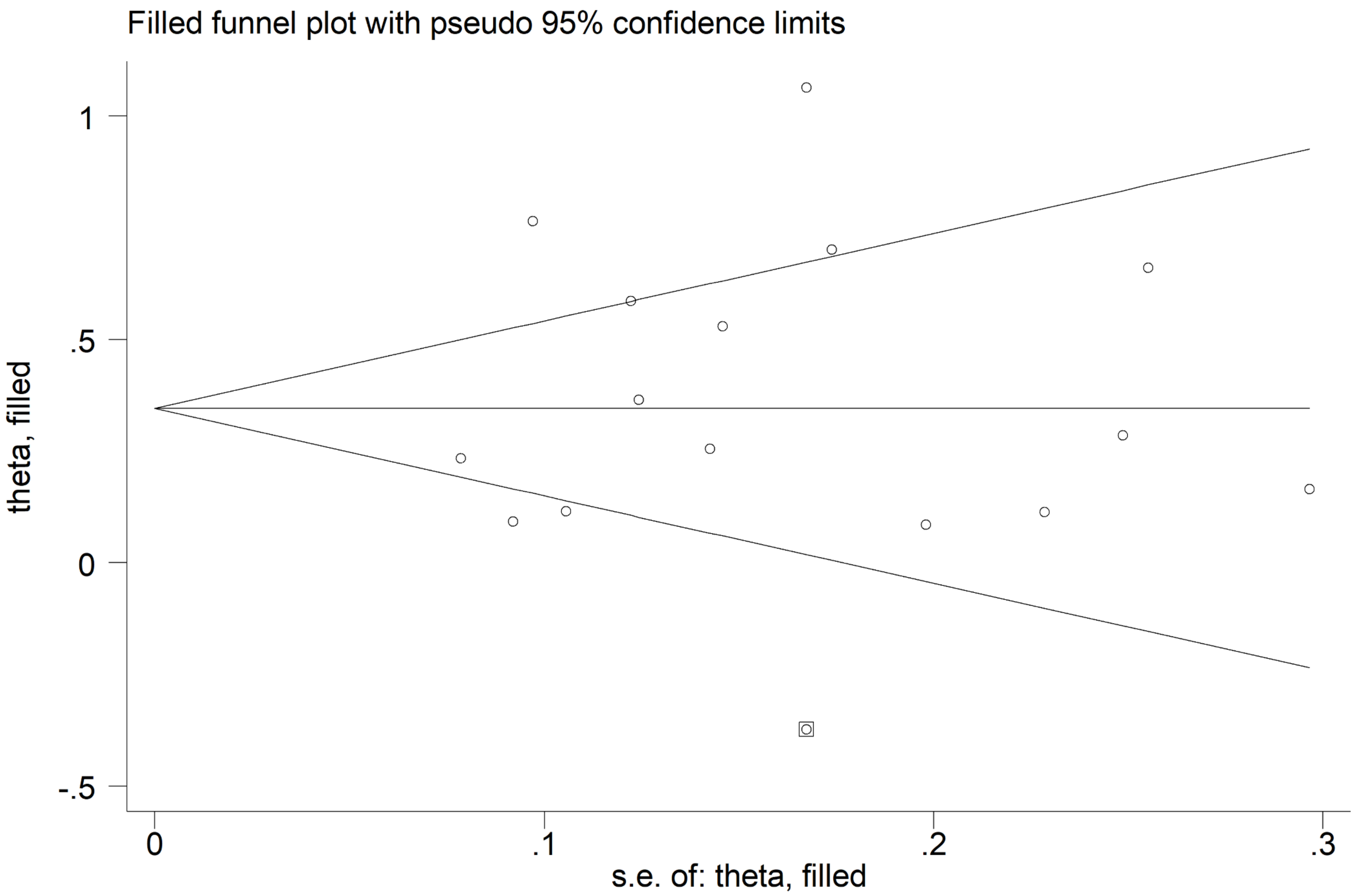


**Suppl. Figure S12.** Funnel plot of studies investigating association between PDW values and OSAS after trimming and filling. Dummy studies and genuine studies are represented by enclosed circles and free circles, respectively.

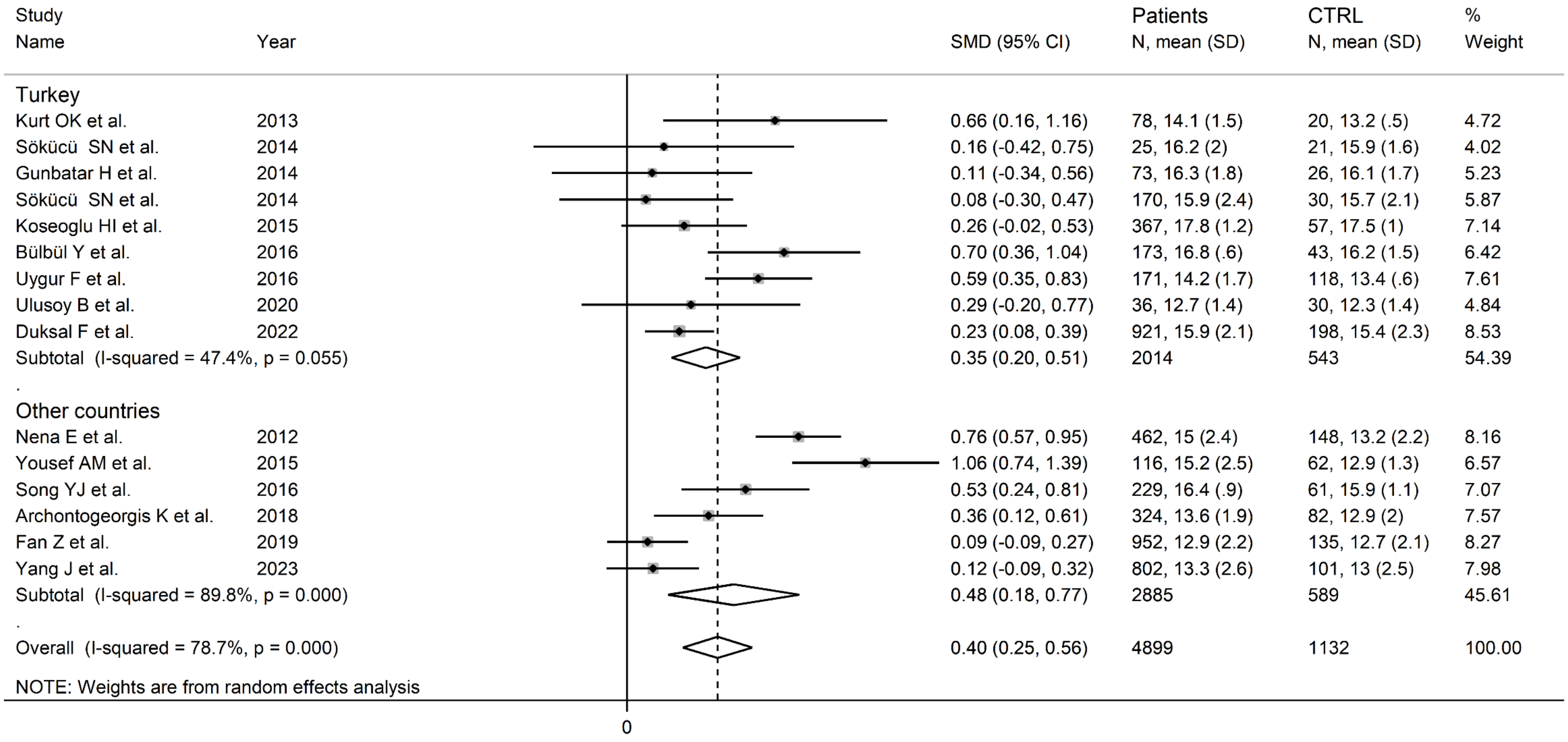


**Suppl. Figure S13.** Forest plot of studies examining PDW values of OSAS patients and controls according to country.

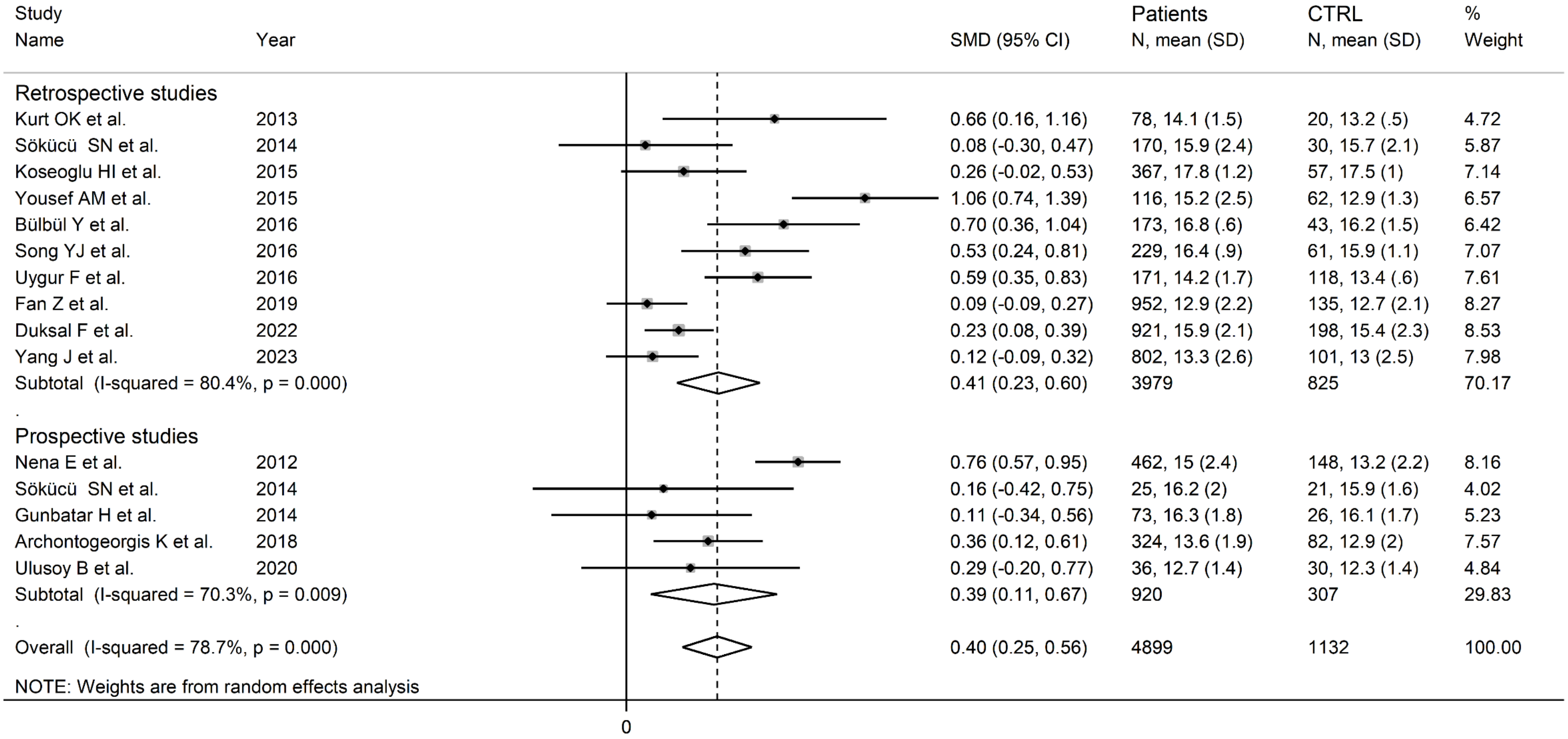


**Suppl. Figure S14.** Forest plot of studies examining PDW values of OSAS patients and controls according to study design.

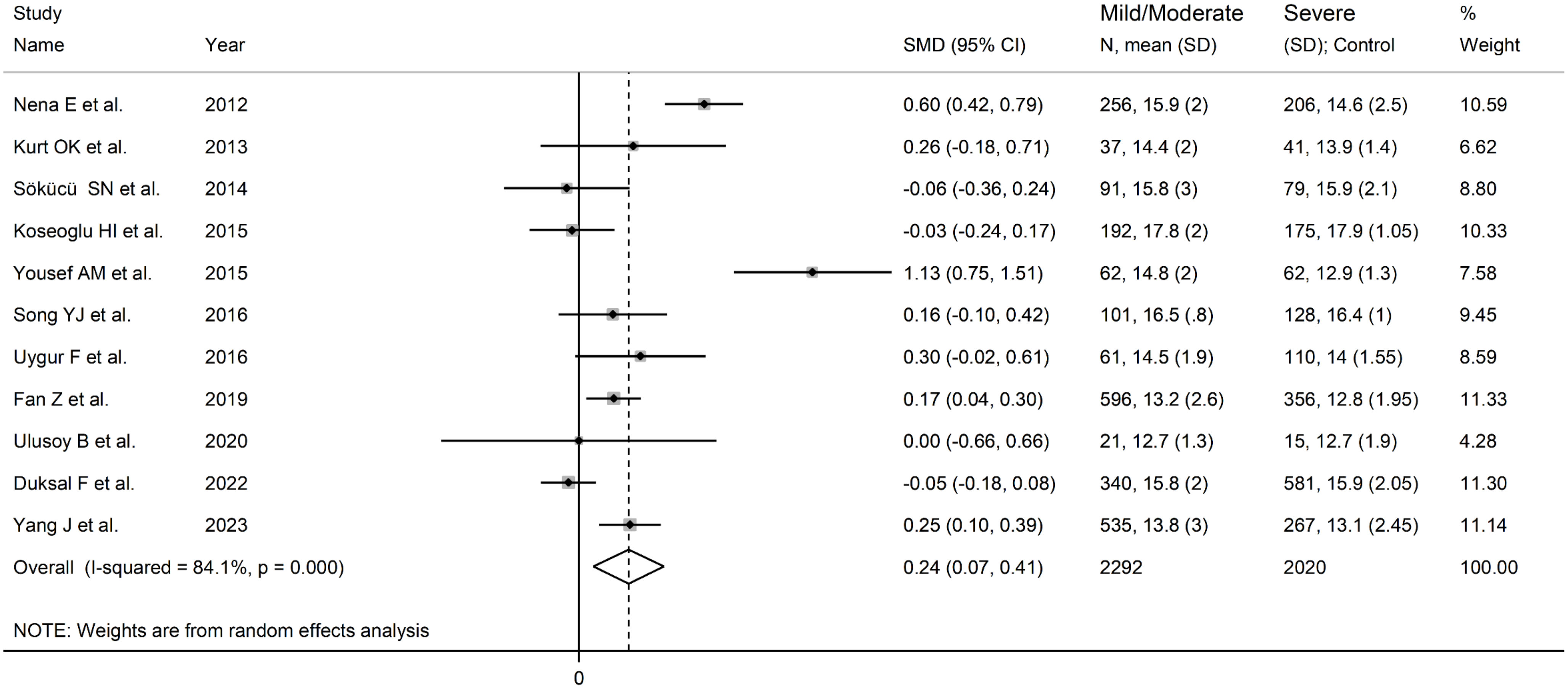


**Suppl. Figure S15.** Forest plot of studies examining PDW values of mild/moderate vs. severe OSAS patients.

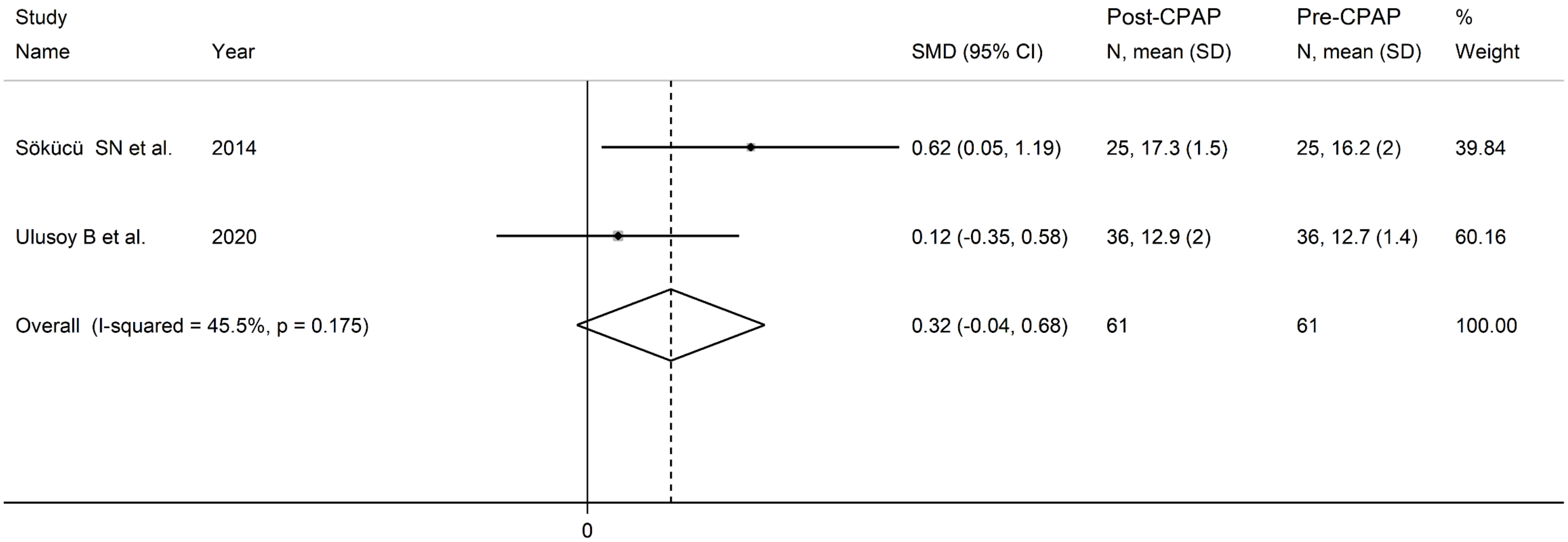


**Suppl. Figure S16.** Forest plot of studies examining PDW values of patients pre- and post-treatment with CPAP.
